# Pediatric HIV Disclosure in Northern India: Evaluation of Its Prevalence, Perceptions amongst Caregivers, and Its Impact on CLHIV

**DOI:** 10.1155/2018/2840467

**Published:** 2018-10-24

**Authors:** Rajesh Meena, Alok Hemal, Shilpa Khanna Arora

**Affiliations:** Department of Pediatrics, Post Graduate Institute of Medical Education & Research and Dr. Ram Manohar Lohia Hospital, Baba Kharag Singh Marg, New Delhi 110001, India

## Abstract

**Background:**

With improving standards of care of children living with HIV (CLHIV), pediatric HIV related mortality rates are declining. New challenges like HIV status disclosure are emerging which need to be addressed to ensure their smooth transition into adulthood. Poor disease disclosure rates are observed in CLHIV globally.

**Aims:**

This study was done to assess the prevalence of HIV disclosure in North Indian CLHIV, know the perceptions of caregivers regarding disclosure, and evaluate the impact of disclosure on CLHIV.

**Methods:**

It was a questionnaire based cross-sectional study carried out amongst 144 caregivers of CLHIV aged 6-16 years attending the pediatric HIV clinic of a tertiary care teaching hospital.

**Results:**

Though the majority (93.8%) caregivers felt that it is important to disclose but only 33% of the children were actually disclosed. Eighty five percent felt that disclosure must be done by one of the family members and correspondingly 73% of the disclosed children were actually disclosed by their parents. Forty seven percent believed that the most appropriate age for disclosure is 10-12 years. The mean age at which disclosure was actually done was 11.06 ± 1.62 years. Comparison of the disclosed and undisclosed CLHIV revealed that the disclosed group had significantly higher age, longer duration of taking ART, and higher proportion of paternal orphans. Age of the CLHIV was the only significant factor for disclosure. Several reasons were cited by the caregivers for nondisclosure. The caregivers observed improved drug adherence in 47.9% of the children following disclosure.

**Conclusions:**

There is a need to develop region specific pediatric HIV disclosure guidelines keeping in mind the caregivers' perceptions. The guidelines must be age appropriate, systematic, and socioculturally acceptable. The most suitable age for disclosure appears to be 10-12 years. Involvement of caregivers and health care providers in the process is a must.

## 1. Introduction

WHO aims to achieve an AIDS FREE generation by the year 2030 and one of the focus groups for interventions is the adolescents living with HIV [1]. Higher rates of loss to follow up and poor drug adherence are important adolescent specific issues that need to be addressed. Disclosure of HIV seropositive status is known to improve adherence and immunological outcomes in children as well as adolescents and hence is a vital step in ensuring a smooth transition into adulthood [2, 3].

HIV diagnosis is no longer a death sentence because of the positive gains from antiretroviral treatment along with a considerable reduction in the incidence of opportunistic infections in children. In spite of these advances, disease status disclosure presents a unique challenge for the health care providers as well as caregivers who find it difficult to carry out, more so with increasing age of the child. Globally, many studies have reported low disclosure rates amongst children living with HIV (CLHIV) ranging from 19 to 33% [4–8]. In order to improve pediatric HIV disclosure rates in a particular region of the world, there is need to understand the locally prevalent beliefs and perceptions of caregivers regarding same. Caregivers are often reluctant to disclose the diagnosis of HIV to their children because of concern regarding the child inadvertently revealing the family's HIV status to others, a perception that the child will not be able to cope with the results, guilt regarding transmission, and belief that the children lack interest in their own health [9]. Caregivers are also concerned about stigmatization, the possibility of children losing their friends and social standing in school [2, 10]. Evaluating the impact of HIV status disclosure on CLHIV might also benefit in relieving caregivers apprehensions and circumvent the barriers to disclosure.

Keeping in mind the poor disclosure rates in the region this study was planned and carried out in a tertiary care teaching hospital in Northern India. The objectives of the study were to assess the prevalence of HIV status disclosure in children and adolescents living with HIV between 6 and 16 years of age (CLHIV); to evaluate the perceptions of caregivers regarding disclosure; and to assess the impact of disclosure on CLHIV.

## 2. Methods

This cross-sectional study was carried out in the pediatric HIV clinic of the hospital from November 2015 to March 2017 after obtaining institutional review board clearance. CLHIV follow up with their caregivers in the clinic which is held once per week (every Monday). In the clinic they are given comprehensive pediatric HIV care including ART. The sample size calculation was done considering the prevalence of disclosure to be around 19% [5]. Taking this value as reference, the minimum required sample size with a 6.5% margin of error and 5% level of significance came out to be 140.

One hundred forty-four caregivers (mother or father or accompanying adult in case of orphans) of CLHIV between 6 and 16 years were consecutively enrolled on Mondays from the clinic after taking written informed consent. An unvalidated questionnaire was used for the interview process. The questionnaire was indigenously developed keeping in mind the available literature and after carrying out a pilot survey of caregivers. All the interviews were carried out by the same investigator to avoid interobserver variability and the number of caregivers interviewed was restricted to 2-3 per recruitment day. The interview was carried out in a separate room ensuring privacy for the interviewee and the responses were recorded in the questionnaire.

The caregivers of CLHIV who lost a family member in the last 3 months or those who were hospitalized with some critical illness were not included in this study. The CLHIV's status was considered as disclosed if he/she knew that he/she was suffering from a chronic illness and was aware of the name of the illness as HIV (full disclosure) [4].

The face to face interview was carried out in English or Hindi language as per the caregiver's convenience and the investigator filled up a detailed questionnaire. The questionnaire inquired about the importance of disclosure and if already disclosed, by whom and at what age was it done. Questions were asked from caregivers about their perception regarding HIV disclosure and the child's HIV disclosure status. In nondisclosed CLHIV, caregivers were additionally inquired about reasons for not disclosing the HIV status of the child. In disclosed CLHIV, caregivers were additionally asked about the impact of disclosure on the child.

Other pieces of information that was collected for each CLHIV were clinical status; sociodemographic profile; HIV status and treatment details of the child as well as caregivers. For the mode of acquisition, clinical and laboratory parameters, anthropometry, and CD4 count values, the clinical records for each CLHIV were reviewed and details recorded. Data was entered into Microsoft Excel spreadsheet and analysis was done using Statistical Package for Social Sciences (SPSS) version 21.0. Categorical variables were presented in number and percentage (%) and continuous variables were presented as mean ± SD and median. Normality of data was tested by Kolmogorov-Smirnov test. If the normality was rejected then nonparametric test was used. Quantitative variables were compared using Mann-Whitney Test between the two groups as the data sets were not normally distributed. Qualitative variables were correlated using Chi-Square test/Fisher's exact test. Univariate and multivariate logistic regression was used to find out the factors significantly affecting HIV disclosure to CLHIV. A p value of <0.05 was considered statistically significant.

## 3. Results

Caregivers of 144 patients who were regularly attending the pediatric ART clinic were interviewed.  Table 1 demonstrates the characteristics of the CLHIV population. The majority of CLHIV (90/144, 62.5%) were in 11-16 years of age group and the rest (54/144, 37.5%) were 6 to 10 years of age. Their mean age was 11.40 ± 2.86 years and the majority (97/144, 67%) were boys. Analysis of data from their clinical records demonstrated that, for most of the CLHIV, fathers (116/144, 80.6%) as well as mothers (128/144, 88.9%) were seropositive. Out of 144 CLHIV, 107 (74.3%) patients' fathers were alive and 99 (68.8%) patients' mothers were alive. The majority of CLHIV were in stage I of the disease (100/144, 69.4%). Vertical transmission was the most common mode of acquisition of HIV (129/144, 89.6%), the rest being blood transfusion (8/144, 5.6%), intravenous drugs (6/144, 4.2%), and unknown in 1.

In this study population, a large number of CLHIV 96/144 (66.7%) were not aware of their HIV status (undisclosed group) and only 48/144 (33.33%) knew of their HIV positive status (disclosed group). The average age at disclosure of HIV positive status was 11.06 ± 1.62 years. Out of 48 disclosed cases, 12 (25%) came to know their HIV status around the age of 10 years. Almost an equal percentage of CLHIV, i.e., 10 (20.8%) and 11 (22.9%), came to know of their HIV positive status around the age of 11 and 12 years, respectively. It was observed that out of 48 disclosed CLHIV, the majority was disclosed by their parents (35/48, 72.9%), 2/48 (4.17%) CLHIV were disclosed by health care providers, and the rest came to know about their illness from other sources (11/48, 22.92%). It was observed that the majority of the CLHIV who came to know about their illness from other sources were paternal orphans (6/11, 54.55%).

Comparison of the disclosed and undisclosed groups revealed that the mean age of the disclosed CLHIV (13.75 ± 1.68 years) was significantly higher in comparison to the undisclosed CLHIV (10.22 ± 2.59 years) (p<0.001). The majority of the CLHIV in the disclosed group were > 10 years old (46/48, 95.83%) and only 2 CLHIV ≤ 10 years were aware of their status. Amongst the undisclosed group, the majority were ≤ 12 years (76/96, 79.17%) but a few CLHIV were aged >12 years as well (20/96, 20.83%). The disclosed CLHIV had been taking ART for a longer duration (5.88 ± 3.06 years) than the undisclosed group (4.43 ± 2.7 years) (p=0.01). The disclosed group had a significantly higher proportion of paternal orphans (18/47, 38.3%) in comparison to the undisclosed group (18/96, 18.75%) (p=0.01). The two groups did not differ significantly in terms of sex, parental (maternal and paternal) HIV status, proportion of maternal orphans, WHO stage, and mode of acquisition of HIV. Logistic regression was performed to assess the factors associated with disclosure (Table 2). Univariate analysis suggested that age (OR=1.97, CI=1.56-2.48, p<0.001), duration of taking ART (OR=1.19, CI=1.05-1.35, p<0.01), and paternal orphans (OR=2.69, CI=1.23-5.87, p=0.01) were significant factors for disclosure. Multivariate analysis revealed that only age of the CLHIV was a significant factor for disclosure (OR=3.33, CI=2.08-5.32, p<0.001).

Table 3 summarizes the perceptions of caregivers regarding disclosure of HIV status to children and adolescents. It was observed that 135/144 (93.8%) caregivers thought that it is important to disclose the child's HIV status to them. The majority (85.4%) felt that one of the family members should disclose the status. Few believed that it is the responsibility of the health professional either alone (12/144, 8.3%) or jointly with caregivers (12/144, 8.3%) to disclose it to the patient. Most of the caregivers (68/144, 47.2%) believed that 10-12 years of age is the most appropriate age for the disclosure of HIV.

The caregivers (96/144) who had not disclosed the HIV status to the children were inquired the reason(s) for the same. The most frequent responses included “the child won't be able to understand about the illness” (89/96, 92.7%), “I didn't disclose as the child may tell the secret to others” (79/96, 82.3%), “the child is too young to understand the disease” (64/96, 66.7%), “child may feel hurt" (44/96, 45.8%), “disclosure may worsen their school performance” (44/96, 45.8%), “it may decrease their drug adherence” (42/96, 43.8%), and “I didn't disclose because of my own illness” (35/96, 36.5%). All the responses have been tabulated in Table 4.

The caregivers of the disclosed CLHIV were inquired about its impact on child's school performance, school attendance, drug adherence, and behaviour.  Table 5 summarizes these findings. Drug adherence improved in 47.9% (23/48) CLHIV following disclosure, 2 (4.1%) CLHIV showed worsening, and the rest 47.9% (23/48) were unaffected.

## 4. Discussion

The present study observed that only 33.33% CLHIV aged 6-16 years were actually aware of their seopositive status. Similar disclosure rates were noted by researchers from Southern Ethiopia (33.3%), Ghana (33%), Tanzania (32.6%), and Latin America (39%) [6–8, 11]. Certain researchers have reported even lower disclosure rates like one from Ghana (21%) and one from Kenya (19.2%) [4, 5]. Two studies carried out in India reported disclosure rates of 14% and 41.4% [12, 13]. According to a systemic review by Vreeman et al. [14], the disclosure rate amongst children and adolescents in resource limited settings ranged from 0 to 69.2%. Such poor HIV disclosure rates reported from several regions of the world are likely to hamper the smooth transition of CLHIV into adolescence and adulthood.

WHO recommends that children of school age (6-12 years) should be told their HIV positive status [2]. Practically, the age range 6-12 years is too wide as the level of understanding of a 12-year-old child would be superior in comparison to a 6-year-old child and hence the strategy to disclose about the disease to the CLHIV aged 6 years apart should be different. The present study attempted to find out the age when the parents would feel more comfortable to communicate with the children about the disease. It was observed that the majority of the parents believed that 10-12 years is the most appropriate age for the disclosure of HIV and the majority of the children were in fact disclosed their status around the same age (mean ± SD = 11.06 ± 1.62 years). The mean age at disclosure reported by a previous Indian study was lower, i.e., 9.1 years [13]. Other researchers have reported the mean age at disclosure to be ranging from 9.2-11 years [8, 9, 13, 15]. Nzota et al. [8] reported the commonest age of disclosure between 10 to 17 years, whereas Vreeman et al. [14] in their systematic review observed that the majority of children between 10 and 14 years were aware of their seropositive status. CLHIV need to adjust to the illness at different life stages, especially while reaching adolescence as they become sexually active and can unknowingly cause disease transmission to partners. Thus many researchers suggest that disclosure must take place before CLHIV reach adolescence and become sexually active [15]. Hence 10-12 years seems to be the most appropriate age for disclosure for this region and is in sync with the WHO guidelines. A significant proportion (21%) of CLHIV in the undisclosed group was aged more than 12 years in this study. Programmatic policies must be made to prioritise such adolescents who must be made aware of their own illness as well as other aspects regarding HIV including transmissibility.

The majority (83%) caregivers in the present study preferred that the person disclosing the seropositive status to children must be the caregivers and this was the case in almost 73% of the disclosed CLHIV in this study population. Other caregivers believed that the health care provider must be involved in the disclosure process either alone or jointly with the caregiver, but actually only 4.17% of the CLHIV were disclosed by the health care providers in the present study. Another Indian study observed that 58% of caregivers felt that the best people to disclose the HIV status to minors are caregivers and family members and only 26% believed that the doctor must be involved in the process [12]. Similarly a study from Ethiopia revealed that 69.8% of the caregivers preferred disclosure to children by family members rather than health professionals [6]. Contrary to this, in a study conducted amongst healthcare workers (HCWs) from South Africa, only half (48.5%) of the HCWs said that the caregivers are the appropriate people to disclose to children and 42.7% felt that disclosure of HIV-infected children is a shared responsibility of the caregivers and the HCWs [16].

A significant finding of the present study is that almost one in five (11/48, 22.92%) of the disclosed CLHIV came to know about their illness from sources other than caregivers and health care providers. One possible reason could be that the majority of these CLHIV were paternal orphans (6/11, 54.55%) and hence were deprived of father's guidance at home. Disclosure from sources other than caregivers and health care providers may have negative implications as unreliable sources may lead to provision of wrong or incomplete information about the disease. Hence there is a need to sensitise and enable caregivers jointly with health care providers to provide disease disclosure at the appropriate age in a positive and systematic way.

Comparison of the disclosed and undisclosed groups revealed that the mean age of the disclosed group was significantly higher and they had been taking ART for a significantly longer duration in comparison to the undisclosed CLHIV. This suggests that older age, longer duration of taking medicines, and frequent visit to the HIV clinic might have prompted CLHIV to inquire about their disease leading to disclosure. The disclosed group also had a significantly higher proportion of paternal orphans as previously discussed. Multivariate analysis revealed that age of the CLHIV was the only significant factor for disclosure in our setting (OR=3.33, CI=2.08-5.32, p<0.001). Similarly, a previous study observed that child's age more than 10 years, duration of HIV diagnosis of 5 years or more and taking a zidovudine based regimen predicted disclosure [6]. A recent study from Latin America also observed that a greater proportion CLHIV were disclosed whose biological father was deceased and the ones who had been prescribed antiretroviral medication [11].

The present study observed that only one in three CLHIV were actually aware about their status in spite of the fact that 93.8% caregivers believed that it is important to disclose the child's HIV status to them. Thus to assess the barriers for disclosure, perceptions of caregivers who had not disclosed the status to their wards were analysed. It revealed that the majority believed that the child was either too young; would fail to understand about illness; or would disclose the secret to others. Many of them felt that disclosure may lead to decrease in drug adherence, worsening of school performance and the child may feel hurt. Few of them accepted that they do not know how to disclose and lacked in depth HIV related information. Similar concerns of caregivers were cited by some previous studies as the reason behind nondisclosure [5–7]. Hence there is a need to develop regional guidelines for pediatric HIV disclosure that are socioculturally acceptable, keeping in mind these parental perceptions and beliefs. The guidelines should be caregiver centric with active involvement of healthcare providers to ensure that the process of disclosure is easier and more acceptable for the CLHIV.

Lack of disease knowledge is known to impact self-care taking behaviour, drug adherence, relationships with friends and community, and future planning. The present study observed that drug adherence improved in 47.9% of the subjects following disclosure. Previous studies have shown that the children who know their infection status perform higher in quality of life assessments and have improved medication adherence [17]. Children who know their status have also been shown to have slower rates of CD4% decline and lower AIDS-related mortality [18].

This study has some limitations. Firstly, the questionnaire used was indigenously devised and not validated. Thus the results of this study cannot be generalized to nonsimilar populations. Secondly the questions on the assessment of the impact of disclosure on CLHIV were asked from the caregivers and not from the subjects themselves and that is too retrospective. Hence the assessment of impact of disclosure may not be accurate.

## 5. Conclusion

This study has attempted to know the caregivers' perceptions and the impact of disclosure on CLHIV at a tertiary care centre in Northern India. Poor disclosure rates observed in the present study indicate that there is an unmet need to develop regional pediatric HIV disclosure guidelines that must be age appropriate and systematic with active participation of caregivers as well as health care providers. The most suitable age for disclosure as perceived by caregivers in the present setting is 10-12 years. As WHO suggests, it is important to tailor the process of disclosure in cultural context; hence the guidelines must be devised keeping in mind the caregivers' perceptions and beliefs. The same principles would hold true for other regions of the world with poor pediatric HIV disclosure rates.

## Figures and Tables

**Table 1:** Patient characteristics.

**Current Age (Years)**	
6 - 10 Years	54 (37.5%)
11 - 16 Years	90 (67.5%)
**Sex of CLHIV**	
Male	97 (67%)
Female	47 (33%)
**Parents' HIV status**	**Father/Mother**
Positive	116 (80.6%)/ 128 (88.9%)
Negative	27 (18.8%)/ 14 (9.7%)
Unknown	1 (0.7%)/ 1 (0.7%)
**WHO Stage**	
I	100 (69.4%)
II	30 (20.8%)
III	09 (6.3%)
IV	05 (3.5%)
**Route of acquisition of HIV**	
Vertical	129 (89.6%)
Blood Transfusion	8 (5.6%)
Intravenous	6 (4.2%)
Other	1 (0.7%)
**Duration of treatment**	
<1 Year	6 (4.2%)
1 - 3 Years	46 (31.9%)
3-5 years	31 (21.5%)
>5 Years	61 (42.4%)

**Table 2:** Univariate and multivariate ordinal regression to assess factors associated with disclosure.

	Univariate Ordinal Regression			Multivariate Ordinal Regression		
	Odds ratio	95% C.I. for Odds ratio	P value	Odds ratio	95% C.I. for Odds ratio	P value
**Age**	1.97	1.56-2.48	<0.001	3.33	2.08-5.32	<0.001
**Duration of taking ART**	1.19	1.05-1.35	<0.01	1.08	0.83-1.40	0.57
**CD4 count**	1.00	1.00-1.00	0.07			
**Sex**						
Female	Reference					
Male	0.95	0.46-1.99	0.90			
**Father's HIV status**						
Negative	Reference					
Positive	0.66	0.28-1.55	0.34			
**Mother's HIV status**						
Negative	Reference					
Positive	0.62	0.20-1.91	0.41			
**Father's living status**						
Alive	Reference					
Dead	2.69	1.23-5.87	0.01	3.86	0.83-17.96	0.09
**Mother's living status**						
Alive	Reference					
Dead	0.80	0.37-1.73	0.57			
**WHO Stage of CLHIV**						
I	Reference					
II	0.91	0.39-2.15	0.83			
III	0.55	0.11-2.78	0.47			
IV	1.28	0.20-8.00	0.80			
**Mode of acquisition of HIV**						
Blood transfusion	Reference					
Intravenous drugs	0.60	0.04-8.73	0.71			
Vertical	1.61	0.31-8.29	0.57			
Unknown	0.00		1.00			

**Table 3:** Perceptions of caregivers regarding disclosure.

**Question**	**Response**
**1. Do you think it is important to disclose his/her HIV positive status to the child?**	
(i) Yes	135/144 (93.8%)
(ii) No	9/144 (6.2%)
**2. Who do you think is the best person to disclose the status?**	
(i) Parents/Caregiver	120/144 (83.3%)
(ii) Health Professional,	12/144 (8.3%)
(iii) Both	12/144 (8.3%)
**3. What do you think is the most appropriate age to disclose?**	
8-10 YR	11/144 (7.6%)
10-12 YR	68/144 (47.2%)
12-14 YR	26/144 (18.1%)
14-16 YR	28/144 (19.4%)
>16 YR	11/144 (7.6%)
**4. Why do you think that that the child must know his/her HIV positive status? (N = 135)**	
(i) Improve drug compliance	114/135 (84.4%)
(ii) For safe sexual behavior	60/135 (44.4%)
(iii) Because it is a chronic disease	62/135 (45.9%)
(iv) Other	35/135 (25.9%)

**Table 4:** Reasons behind nondisclosure of HIV status.

**S. No.**	**Reason**	**Frequency (Percentage)**
1.	Child won't be able to understand about the illness	89 (92.7%)
2.	He/ she might tell the secret to others	79 (82.3%)
3.	Child is too young to understand	64 (66.7%)
4.	Child's school performance may worsen	44 (45.8%)
5.	Child may feel hurt	44 (45.8%)
6.	Disclosure may decrease his/ her adherence	42 (43.5%)
7.	I didn't disclose because of my own illness	35 (36.5%)
8.	I lack in dept HIV related information	9 (9.4%)
9.	Health professional should disclose	7 (7.3%)
10.	I don't know how to disclose	4 (4.2%)
11.	I avoid thinking about my illness	1 (1.0%)

**Table 5:** Impact of disclosure on children.

**Parameter (N=48)**	**Improved**	**Worsened**	**No change**
**School performance **	5 (10.4%)	3 (6.3%)	40 (83.3%)
**School attendance **	5 (10.4%)	3 (6.3%)	40 (83.3%)
**Drug adherence **	23 (47.9%)	2 (4.1%)	23 (47.9%)
**Behavior **	6 (12.5%)	7 (14.6%)	35 (72.9%)

## Data Availability

The data used to support the findings of this study are included within the article.
